# Factors associated with acceptability of HIV self-testing (HIVST) among university students in a Peri-Urban area of the Democratic Republic of Congo (DRC)

**DOI:** 10.11604/pamj.2018.31.248.13855

**Published:** 2018-12-27

**Authors:** Ben Bepouka Izizag, Hippolyte Situakibanza, Taty Mbutiwi, Richard Ingwe, Florian Kiazayawoko, Aliocha Nkodila, Madone Mandina, Murielle Longokolo, Evelyne Amaela, Marcel Mbula

**Affiliations:** 1 Division of Infectious Diseases, University Teaching Hospital of Kinshasa, Kinshasa, Democratic Republic of Congo; 2 Faculty of Medicine, University of Kikwit, Kinshasa, Democratic Republic of Congo; 3 National Program of HIV/AIDS and STD, Kinshasa, Democratic Republic of Congo; 4 Division of Internal Medicine, Hospital of Boma, Kinshasa, Democratic Republic of Congo

**Keywords:** Acceptability, HIV self-test, students

## Abstract

**Introduction:**

this paper examines the acceptability of HIV self-testing (HIVST) by students in a university in the DRC and identifies factors associated with uptake of HIVST.

**Methods:**

a cross-sectional study was conducted with a sample of 290 students from Kikwit University. Data were summarized using proportions and predictions of acceptability of HIVST by logistic regression.

**Results:**

the average age of students was 22.5 years, with the majority of the students being male (57%). Just over half the students sampled, reported being sexually active (51.8%). One hundred and sixty four (75%) reported that they had one sexual partner and fifty-six (25%) two or more sexual partners in the past year. Sixty-six percent had used condoms during their last sexual encounter. The acceptability of HIVST was high (81.4%) and 66.1% of students stated that they would confirm the self-test at a local health facility. The knowledge about the importance of the self-test (OR 5.02; 95% CI:1.33-18.88; p=0.017), the perception that counseling pre and post-test were important (OR 2.91; 95% CI:1.63-5.19; p < 0.0001) and the willingness to realize the test with a partner (OR 2.46; 95% CI:1.43-4.23; p=0.034) were factors associated with HIVST.

**Conclusion:**

the acceptability of HIVST was high and therefore its implementation is feasible in our country. However, prior to implementation, additional factors such as cost; access of HIVST; false reassurance of the test; missed early infections in the window period, limited counseling and linkage to care options, need to be considered.

## Introduction

In recent years, there has been increasing optimism around achieving an AIDS-free generation [1]. The Joint United Nations Program on HIV/AIDS has outlined its ambition for supporting such an AIDS-free generation, by establishing a series of goals to this end. These goals can be summarized as the UN HIV 90-90-90 program. This program aims to ensure that 90% of people living with HIV/AIDS (PLWHA) know their status, to initiate 90% of these individuals on treatment and to achieve a viral load suppression in 90% of this group [2]. Reaching these targets will require bolstering the entire cascade of HIV care, which in turn, starts with getting more people tested for HIV [3, 4]. In an effort to achieve the United Nations (UN) 90-90-90 global HIV target and specifically the first target of diagnosing of 90% of all people with HIV, the World Health Organization (WHO) released the consolidated guidelines on HIV testing services in 2015. Therein, they highlighted the potential of HIV self-testing (HIVST) to increase HIV testing service (HTS) access, especially among men, key populations and young people. They also highlighted the need to improve the uptake of couples and partner testing services, including offering HTS to the partners of people with HIV [5]. Young people, aged between 15 and 24, are particularly vulnerable to both acquiring and transmitting HIV and more than 50% of all new infections worldwide are among young people between within this age group [6]. The HIV test was developed in mid-1980 and was intended to be accompanied with HIV counseling [7]. However, with the growing awareness of HIV/AIDS and the recent availability of antiretroviral therapy (ART), the scope and reasons for voluntary counseling and testing (VCT) have broadened. These services are essential components of HIV prevention and care programs. In the past many people were reluctant to be tested even if the care program and treatment were available to them [8]. Thus, HIVST has the potential to facilitate the testing of more people, because it has advantages of convenience, speed, privacy and accessibility, thus eradicating many of the barriers that prevent people from being tested. It also has the potential to be an acceptable option for high risk populations who would not otherwise be tested using currently available HIV counseling and testing (HCT) services for various reasons [9]. The adoption and implementation of HIVST as a testing option in many African countries as well as globally does however come with challenges. Health experts and government stakeholders have criticized the technology for its risk of inaccurate results, potential psychological risk posed by a lack of adequate counseling and uncertainty over unsupervised linkage to care for individuals who test positive for HIV [10-12]. Here we have conducted a study to assess the implementation of HIVST intervention among young people in the DRC. The objective of this study was to evaluate the acceptability of HIVST and identify factors associated with HIVST among students in Kikwit University.

## Methods

**Study area and study period:** this study was conducted from March to April 2017 in Kikwit University.

**Study design:** cross sectional study design.

**Study population:** all current students of Kikwit University were considered as the study population.

**Study setting and sample:** an institution based cross sectional study was conducted in Kikwit University, which was founded in 1992 and is located in Kikwit city in the Kwilu Province, a peri-urban area 500 KM from Kinshasa, in the South-west of the Democratic Republic of Congo. It is one of the prestigious public institutions. The university has four departments (faculties) which are medicine, law, agronomy and economics with a total of 2525 students enrolled.

**Sampling:** the sample size was computed by using the following formula:

$$n=za^{2}pq/r^{2}$$

where p represents the proportion of acceptability to HIVST, taking the proportion of 87.1% [13] and considering a 10% non-response rate, q (1 - p) and z, the value of the standard normal distribution corresponding to a significance level of alpha of 0.05 (1.96). We assumed, d (the precision degree) to be 5%. Using the equation we calculated a minimal sample size of 208 students. A total of 290 students were selected by using a stratified sampling technique and the number of study participants for each selected department was obtained from the registrar's office. The total sample size was allocated proportionally according to the size of all four selected faculties and then simple random sampling was employed to select students from each faculty. The students were asked to fill out the questionnaire at their leisure.

**Exclusion criteria:** students who studied in the evening were excluded from the study.

**Instrument of data collection and techniques:** data was collected by using a self-administered structured questionnaire which was printed in French. The study's instruments were the self-administered and structured questionnaire that included questions on socio-demographic background, sexual behavior, HCT practices and opinions and the acceptability of HIVST. We used a validated questionnaire from the Australian and South African Students and Sexual Health Survey [13, 14]. During our study, Students were asked about whether they would use HIVST, their willingness to buy self-testing kits, their opinions on the provision of the pre- and post-testing for HIVST and also about the information on how to use self-testing kits. For evaluating the acceptability and opinions of the students about HIVST, we asked students if they were aware of HIVST, whether they would like to start using HIVST as a testing option, whether pre and post-test counseling was necessary for HIVST, and also if they were willing to buy a self-test kit. We also asked them if they would encourage their partner to do the HIVST and if they would submit the test results for statistical purposes. Additionally we asked if they would confirm a positive test result by going to a local clinic. All test results, whether positive or negative have been submitted for collating with the HCT statistics.

**Data collection and quality control:** data collection was conducted by four general practitioners. Data collectors received a half day training in using the questionnaires (topics included: the objective of the study, how to approach the participants, how to administer and collect the questionnaires on the time). Consequently, the questionnaire was revised, taking into account the advice of the practitioners, before data collectors were disseminated to collect data. Confidentiality of the study participants was maintained during distribution and data collection periods. Above all, ethics, coding and entry were maintained throughout the process.

**Data analysis:** prior to data entry, the questionnaires were checked for completeness. Partially completed questionnaires were excluded from analysis. The questionnaires were coded and the data entered into Excel and analyzed by the SPSS Version 21 package. Collected data was summarized with the use of frequency, percentage, and odds ratios. Binary/multiple logistic regression analyses were used in order to assess the association and measure the level of significance. Further logistic regression was used to adjust for possible confounding factors and the results were reported by using P < 0.05 level of statistical significance.

**Ethical considerations:** ethical approval was obtained from the Ethics committee of the University of Kinshasa. Consent was requested and obtained from each student prior the study. No personal identification was recorded on the questionnaire for ethical reason.

## Results

**Demographics, sexual behaviors and the HCT opinions of the students:** of the two hundred ninety students who participated in this study, 57% were male and 43% female. The average age of the students was 22 years (age range 19±33 years). Most of the students (n = 154.63) were between 20 and 24 years old. Half of the students (51.8%) reported regular sexual activity. 74.5% reported having only one partner, 16.4% two partners and 9.1 % more than two partners. 52% of students reported the use of condoms when having sex. A total of 46.9% of the participants had never been tested for HIV, while 39.9% of students reported that they knew the HIV status of their sexual partner/s. The majority of the participants, 95.5%, considered HIV testing to be important for young people and 92.5% said that HIV pre-test counseling was necessary (Table 1).

**Table 1:** demographics, sexual behaviors and the HIV counseling and testing (HCT) opinions of the students

Variables	Frequency (N)	Percentage
**Gender (missing value=3)**		
M	164	57.1
F	123	42.9
**Religion (missing value=21)**		
catholic	157	58.4
protestant	38	14.1
Revival churches christian	53	19.7
kimbanguist	5	1.9
other	16	6.0
**Sexually active (the missing value=18)**		
yes	141	51.8
no	131	48.2
**Have a current sexual partner**		
yes	199	68.9
no	91	31.1
**Ever tested for HIV ( missing value=2)**		
yes	151	52.4
no	135	46.9
Not sure	2	0.7
**Number of sexual partners in the past year (missing value=70)**		
One	164	74.5
Two	36	16.4
More than two partners	20	9.1
**Condom use during last sexual encounter (missing value=44)**		
Yes	128	52.0
No	117	47.6
Not sure	1	0.4
**Know HIV status of sexual partner (missing value=42)**		
Yes	99	39.9
no	128	51.6
Not sure	21	8.5
**HIV testing important for young people (missing value=46)**		
yes	233	95.5
no	8	3.3
Not sure	3	1.2
**Pre-test counseling for HIV necessary (missing value=49)**		
yes	223	92.5
no	17	7.1
Not sure	1	0.4

**Opinions and acceptability regarding HIVST:** in the present study, 54.5% of the participants were aware of HIVST before the survey. The acceptability of HIVST as a testing option was 81.4%. The proportion of students who indicated the necessity for pre-test counseling for HIVST was 70.4%. Seventy three percent of students agreed on the necessity for post-testing kit counseling for the HIVST and 78. 4% expressed a willingness to buy self-testing kits themselves. Sixty eight point four percent of students agreed to use HIVST with their partners. We also found that 55.5 % were willing to submit the HIVST results to a local clinic for inclusion in the HCT statistics. The target of the HCT uptake campaign was 55.5%. The willingness to confirm a HIV positive self-test result at a local health care facility was 66.1% (Table 2).

**Table 2:** opinions and acceptability regarding HIV self-testing (HIVST)

Variables	Frequency (N)	Percentage
**Ever heard of HIVST prior to survey (missing value=13)**		
Yes	151	54.5
No	126	45.5
**Pre-test counseling is necessary for HIVST(missing value=10)**		
Yes	197	70.4
No	72	25.7
Not sure	11	3.9
**Post-test counseling is necessary for HIVST(missing value=15)**		
yes	201	73.1
no	71	25.8
Not sure	3	1.1
**Willingness to buy the self-testing kit (missing value=7)**		
yes	222	78.4
no	53	18.7
Not sure	8	2.8
**Willingness to use HIVST if it was available (missing value=37)**		
Yes	206	81.4
No	41	16.2
Not sure	6	2.4
**Test with a partner by using HIVST kits (missing value=5)**		
Yes	195	68.4
No	79	27.7
Not sure	11	3.9
**Willingness to confirm HIVST results in a formal health care facility (missing value=4)**		
Yes	189	66.1
No	85	29.7
Not sure	12	4.2
**Willingness to submit HIVST results for health statistics (missing value=9)**		
Yes	156	55.5
No	107	38.1
Not sure	18	6.4

**Factors associated with the uptake of HIVST:** in order to determine factors associated with uptake of HIVST among students in the University of Kikwit, multivariate logistic regression analyses were performed. Independent variables included demographic (age and gender), sexual behavior (sexual relationships, number of sexual partners and use of condoms), HCT practices and opinions of the students (having been tested for HIV before, knowledge of HIV status of their sexual partners, knowledge of the importance of the HIV test, the necessity of HIV pre-test counseling, the willingness to submit HIVST results at a local clinic for inclusion in the HCT statistics, to confirm HIV positive self-test results at a local health care facility, the willingness to buy a HIVST and to share' an HIVST with their partner). These factors were compared against the outcome variables in a multivariate linear regression model. The acceptability of HIVST in this model, the knowledge of the importance of the test (OR : 5.02 95% CI:1.33-18.88, p=0.017), and perception of counseling the pre and post-test are important (OR : 2.91 95% CI : 1.63-5.19, p < 0.0001) were calculated. The willingness to realize the test with a partner (OR: 2.46 95% CI:1. 43-4. 23, p=0.034) was another factor associated with HIVST (Table 3).

**Table 3:** factors associated with the uptake of HIV self-testing (HIVST)

	Univariate analysis		Multivariate analysis	
Factor	p	OR (95% CI)	p	ORaj ( 95% CI)
**HIV testing important for young people**				
No	0.006	1	0.017	1
yes		6.13(0.27-17.08)		5.02(1.33-18.88)
**Pre-test counseling is necessary for HIVST**				
No	0.008	1	<0.001	1
yes		3.37 (0.61-3.11)		2.91(1.63-5.19)
**Willing to buy the self-testing kit**				
No		1		1
yes	0.030	13.3 (4.87-36.29)	0.320	11.07 (5.20-23.56)
**Test with partner using HIVST kits**				
No		1		1
yes	0.007	3.18 (0,51-2,73)	0.001	2.46 (1.43-4.23)
**Willing to submit HIVST results for the health statistics**				
No		1		1
yes	0.553	2.26 (0.59-2,70)	0.234	1.79 (1.04-3.06)

## Discussion

According to the consensus statement from the first international symposium on HIVST, despite a lack of data regarding its effect on populations, HIVST has a vast potential to scale-up access to HTC services [15]. However, before promoting it widely, the WHO/UNAIDS meeting highlighted the need for evidence-based studies on the potential implementation of HIVST programs in various settings. Our study is a contribution towards this goal by providing data on acceptability of HIVST as well as the demographic and behavioral factors that are associated with the uptake of HIVST among students in the DRC. This study assessed the students' HIV testing practices, using the available HCT services, the acceptability of HIV self-testing and other associated factors among students in Kikwit University before the implementation of this strategy in the DRC. Half the students in this study had had a sexual relationship and 33.3% of these reported having multiple sexual partners. Forty eight percent did not use a condom in their last sexual encounter. This result matches findings of Mogkatle *et al.* in South Africa. Contrary to Mogkatle's results, half of the students in the Kikwit sample knew their serologic status. This result was lower than the finding of Mogkatle *et al.* (72%). This may be explained by the difference between a Southern African versus Central African setting and the fact that Kikwit is a peri-urban site while the South African study was conducted in an urban site [13]. Our data suggests that there is a high frequency (81.4%) of acceptability of HIV testing among students of Kikwit and 68% of them would take up HIV self-testing with their respective partners. The high HIVST acceptability has also been illustrated in different population groups in other studies in Sub-Saharan countries [13, 16-18]. It is possible that with the roll-out of a HIVST program, the frequency of HIV testing among students could therefore increase. At the international symposium on HIVST [15], a concern was raised that the frequency of HIVST alone should not be used as a preventive strategy. The WHO has highlighted the importance of the message that HIVST does not provide a verified diagnosis on HIV, but requires further testing. Therefore despite its ease of use and high acceptability, illustrated in this study, HIVST should contain clear instructions for the use and interpretation of results, as well as how to access the HIV prevention, care and treatment services [19]. Based on the models assumptions, we realize that the lower titers of antibodies and the longer window period of the available oral HIVST kit have more false negative results than facility-based HTC [20]. In terms of confirming positive test results, more than half of the students (66 %) reported their intention to confirm their HIV positive self-test at a local health care facility. This result was lower than that found by Mogkatle *et al.* in South Africa (75%) and higher than that found by Kurth *et al.* in Kenya (35.5%) [13, 21]. In South Africa, the awareness raised about HIV infection is higher than the DRC. It has been recommended that self-testing be in some way linked to a health care facility so that diagnosis can be associated with confirmation and treatment. However currently, there are no reported examples of referral mechanisms to link people who self-test to treatment and care [10].

Van Royen.H. and his colleagues [8] suggested that the self-testing instruction kits should provide adequate information on what to do after self-testing. In our study we did not include any questions regarding instructions in the test kit. It is possible that if interviewers had explained that the kit would instruct a person on what do after testing that less than 34% of the students would have reported no intention to confirm their self-test results. It is important that the provision of HIVST, as with all HTC services, includes programming and messaging about how to access and link a patient to HIV prevention, care and treatment services. There is no doubt that the pre- and post-test counseling remains a challenge for the scaling up of HIVST. The accessibility of services must complement the convenience of HIVST. This could be achieved by toll-free hotlines for example. This will be vital for the scaling up and implementation of HIVST. A number of factors which were associated with the uptake of HIVST among these students, such as: the knowledge of the importance of the test, their perception of pre and post-test counseling and the willingness to take the test with a partner are recognised as important indicators of HIV awareness and prevention. Regarding the knowledge of the importance of the test, HIV counseling and testing (HCT) are among the most effective and important interventions for managing the HIV epidemic [22]. There is substantial evidence showing the link between increased HCT and reduced HIV incidence [23, 24]. One of the problems of existing HCT programs leading to a lack of knowledge of HCT is that leaners are only counseled once they make the decision to test. By including HCT, the pre-test counseling can take place sooner within a general health setting before a decision to test is taken. The increase in knowledge and confidence in the HCT process is likely to act as an individual facilitator. In reality, while counseling is offered to everybody who undergoes HCT, counselors may have different levels of training, and have different attitudes towards clients [25]. Their perception that counseling pre and post-test is important, is itseld a critical factor. This was the second factor associated to acceptability of HIVST in our study. In the case of adolescents, counseling has a wider value; as it is a phase of life in which new skills and abilities are acquired which con¬tribute to ensuring self-care in health throughout adulthood. The diagnosis of a chronic and incurable disease like AIDS in adolescence has a greater impact, since it may cause several changes in the daily life, in the student life and in the process of socialization outside the family environment [26]. From the psychological point of view, adolescence coincides with the first realization of loss and grief [27]. When an adolescent has a serious illness in this period, these losses and grief will be much more painful and difficult to overcome, and the experience of the disease can bring greater suffering. In this sense, the man¬ner in which the pre- and post-HIV test counseling is executed is of vital importance and can increase or reduce the impact of diagnosis and treatment [28]. The third associated factor was the willingness to take the test with a partner. HIV self-testing is a novel strategy that is attractive to both women and men and may prove better at reaching male partners than many other current methods. By its very nature, HIVST is more flexible and autonomous than other forms of HTC and therefore may provide an alternative avenue to the narrow definitions of couples-HTC. Gender and power relations will continue to shape the different stages of decision making, but the ability to discretely determine the circumstances and timing upon which a test is take has been shown to be empowering. This may enable women to influence domestic decision-making without provoking negative reactions from their male partners [29].

## Conclusion

Most of the students in this study freely accepted HIVST and agreed to encourage their partner to do the test as well. The knowledge of the importance of the test, perception of counseling the pre and post-test and the willingness to take the test with their partner were factors associated with HIVST. The results suggest that the implementation of the test is feasible in our country, but still premature because of concerns such as the cost, access of HIVST, the possibility of false negative test results and missed early infections in the key window period. Additionally limited counseling and linkage to care options are factors that will also need to be addressed, should this strategy be implemented.

**Limitations:** the limitations of this study are that we cannot assume causality of the statistically significant associations with acceptability of HIVST in this study given its cross-sectional design. It is possible that unknown or unmeasured factors could have confounded the estimates of the observed associations in our results. The data show a high proportion of willingness to use HIVST but a low linkage to care after self -testing. Some questionnaires were not answered in full, which lead to missing data points; however the sample size in the analysis was adjusted to account for this problem.

### What is known about this topic

- HIV self-testing (HIVST) is globally accepted as an important complement to existing HIV counseling and testing (HCT) approaches;
- The acceptability of HIVST among students and the population groups with poor uptake of HCT remain limited in Africa;
- HIVST can be performed accurately and it is an acceptable and feasible testing approach in a variety of contexts; including populations at ongoing HIV risk and those who may not otherwise be tested.

### What this study adds

- Half of students interviewed were sexually active but the use of condom was not high despite a high desire to use the HIVST;
- A majority of students had the will to confirm the result of a HIVST in a local health care facility, which reinforces the link between the patients and the health system;
- The implementation of the HIVST is not possible now in our country taking into account issues related to accessibility and false belief of a negative result within the window period.

## Competing interests

The authors declare no competing interests.
